# A meta-analysis of the incidence rate of postoperative acute kidney injury in patients with congenital heart disease

**DOI:** 10.1186/s12882-020-02005-2

**Published:** 2020-08-17

**Authors:** Dandan Li, Zhaozhuo Niu, Qiang Huang, Wei Sheng, Tianyi Wang

**Affiliations:** 1 Department of Environmental Health, Qingdao Centers for Disease Control and Prevention, Qingdao, 266033 Shandong China; 2 Department of Cardiovascular Surgery, Qingdao Municipal Hospital, No. 5 Donghai Middle Road, Shinan District, Qingdao, 266000 Shandong China

**Keywords:** Acute kidney injury, Congenital heart disease, Meta-analysis, Incidence rate

## Abstract

**Background:**

Acute kidney injury (AKI) is a common complication of cardiac surgery. However, the incidence rate of AKI in patients with congenital heart disease (CHD) greatly varies between reports owing to the different definitions used for AKI. Therefore, this study was designed as a meta-analysis aimed at summarizing the incidence rate of AKI in patients with congenital heart disease (CHD) on the basis of different AKI criteria.

**Methods:**

Studies published till April 24, 2020, on the incidence rate of AKI in patients with CHD, were retrieved from electronic databases and printed literature. To pool data from the included studies, the effect size, a combined statistics, was chosen and presented with the incidence rate and 95% confidence interval (CI). Heterogeneity was evaluated using *I*^2^ statistics and Cochran *Q* test. The incidence rates obtained from the subgroup analysis according to study location, type of surgery, type of cohort, age, and AKI criteria) were also evaluated to determine the correlation of AKI with these factors. Publication bias was estimated using the Egger test.

**Results:**

Thirty studies, comprising 9925 patients with AKI who had CHD, were included. Overall, the pooled incidence rate of AKI in the patients with CHD was 38.4% (95% CI, 32.0–44.7%). However, the incidence rate was not significantly affected by gender, study location, type of surgery, type of cohort, and AKI criteria. Moreover, age was significantly associated with the incidence of AKI, and the incidence rate was higher in the patients aged < 1 month than in those aged 1 month to 18 years, < 18 years, and ≥ 18 years (*P* < 0.05).

**Conclusions:**

In this study, the estimated incidence rate of AKI in patients with CHD was 38.4% and may be influenced by age. These findings highlight the importance of further investigation of the specific causes of and effective preventive measures for AKI.

## Background

Congenital heart disease (CHD), the most common type of congenital birth defect, is the major cause of mortality among children [1, 2]. The prevalence rate of CHD is estimated to be 8 cases per 1000 live births [3]. An estimated 20% of patients may require surgery during the first 15 years of adulthood, of whom 40% would need reoperations [4]. Despite the marked improvement in survival after cardiac surgery for CHD, the mortality rate in children with CHD treated with after cardiac surgery is 4% [5]. In addition, in adults, the associated mortality rate increases up to 6 months (2.4%) after surgery, with an overall 30-day mortality rate of 1.5% [6].

Postoperative acute kidney injury (AKI), a common complication of cardiac surgery [7], is a clinical syndrome characterized by water, electrolyte, and acid-base imbalances, and accumulation of nitrogenous wastes caused by a rapid decrease in glomerular filtration function within hours to weeks [8]. AKI occurs in 2.7–70% of patients with CHD who undergo cardiac surgery [9, 10]. The inconsistent incidence rates of AKI in patients with CHD may be associated with the differences in patient population, sample size, and AKI identification and classification criteria among studies. In addition, AKI may cause prolonged mechanical ventilation and hospital stay and high morbidity and mortality rates [11–14]. Several previous systematic reviews and meta-analyses mainly focused on the incidence rate of AKI in patients who had undergone total hip arthroplasties [15] or cardiac transplantation [16], but no meta-analysis of pooled incidence rates of AKI in patients with CHD from relevant studies has been reported yet.

Currently, different AKI identification and classification criteria are used in different clinical studies [17, 18]. Initially, the AKI Network (AKIN) criteria, also known as the modified pediatric Risk, Injury, Failure, Loss, End-Stage Kidney Disease (pRIFLE) criteria, were developed on the basis of the pRIFLE criteria with the addition of serum creatinine (SCr) level [19]. The Kidney Disease Improving Global Outcomes (KDIGO) was then proposed, by combining the RIFLE and AKIN criteria, to determine the SCr level and urine output [20]. Studies that reported the epidemiology of AKI greatly varied owing to the different definitions used, and the observations on the incidence rate of AKI were difficult to compare [18]. Thus, this meta-analysis was retrospectively performed to summarize the incidence rate of AKI based on the three AKI criteria in patients with CHD.

## Materials and methods

### Search strategy

PubMed (http://www.ncbi.nlm.nih.gov/pubmed), Embase (http://www.embase.com), and The Cochrane library (http://www.cochranelibrary.com) were chosen for systematic literature retrieval according to the pre-established search strategy. The retrieval keywords included “Acute Kidney Injury,” “AKI,” “Acute Renal Failure,” “ARF,” “Congenital Heart Disease,” “CHD,” and “Congenital Heart Defects.” The retrieval time was up to April 24, 2020, without language restrictions. Detailed information on the retrieval steps and results of the database search on PubMed (Supplementary Table 1), Embase (Supplementary Table 2), and The Cochrane Library (Supplementary Table 3) were provided. Furthermore, a manual search for conceivably related studies using the references of the included articles was additionally performed.

### Study selection criteria

The following types of studies were included: (1) those that included subjects with CHD who underwent cardiac surgery without a history of kidney transplantation; (2) those with the incidence rate of AKI as research outcome; (3) those that used the pRIFLE, AKIN, and KDIGO diagnostic criteria for AKI; and (4) prospective or retrospective cohort studies.

The following types of studies were excluded: (1) reviews, letters, or comments, and (2) those that included the latest or most complete information among repeated or multiple studies that used the same data.

### Data extraction

Two investigators (Dandan Li and Zhaozhuo Niu) independently screened the eligible studies using a predesigned data collection form. The following information was included: first author’s name, publication year, research region, recruitment time, age, sample size, research type, operation type, and AKI definition. After data extraction, any discrepancies were resolved via discussion with two other investigators (Qiang Huang and Wei Sheng).

### Assessment of methodological quality

The Agency for Healthcare Research and Quality-recommended scale was applied for the evaluation of the methodological qualities of the included studies [21]. This scale evaluates studies on the basis of an 11-item checklist, with a final score ranging from 0 to 11 points. Studies with scores < 4, between 4 and 7, and > 7 were considered as having low-, moderate-, and high-quality methods, respectively.

### Statistical analyses

All the meta-analyses were performed using Stata version 10.0 (Stata Corporation, College Station, TX, USA). Incidence rates with their 95% confidence intervals (CIs) were used to evaluate the effect value. The heterogeneity among studies was calculated using the Cochran *Q* test [22] and *I*^2^ test [23]. When the *Q* statistics *P* value was < 0.05 or *I*^2^ value was > 50%, a statistically significant heterogeneity among studies was considered, and a random-effects model of meta-analysis was used to pool the estimates. Otherwise, a fixed-effects model was used. A subgroup analysis was performed according to study location, type of surgery, type of cohort, age, and criteria of AKI to evaluate the correlation between these factors and the study outcome. In addition, the publication bias in the meta-analysis likely led to small-study effects, where the smaller studies might have shown larger treatment effects. The Egger test is a statistical test for small-study effects that may detect increased bias with increasing numbers of trials on the basis of a linear regression model [24, 25]. In this study, publication bias was assessed using the Egger test. *P* values of < 0.05 indicated statistical significance.

## Results

### Literature retrieval

**Fig. 1:**
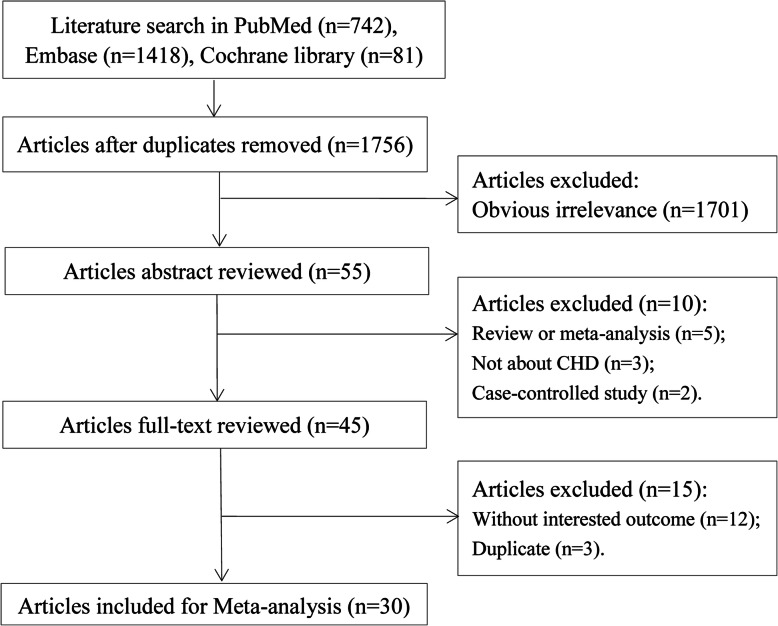
Flowchart of literature search and study selection

The literature retrieval results and screening process are shown in Fig. 1. A total of 2053 citations were generated from PubMed (*n* = 742), Embase (*n* = 1418), and the Cochrane Library (*n* = 81). Among the articles, 485 duplicate studies and 10 reviews or meta-analyses not related with CHD and case-control studies were excluded. Thus, 45 articles remained after a full-text review. Furthermore, 15 articles were excluded because they did not have the outcome of interest or were duplicate studies. Finally, 30 articles [9–13, 26–50] were selected for the meta-analysis.

### Characteristics of the studies

Table 1 shows the characteristics of the 30 included studies published from 2011 and 2020 and distributed in China, Japan, the United States, and Canada. The sample size of the included studies was 9925 patients (range, 47–1510 cases), in which 3506 patients (range, 4–503 cases) had AKI. Among the 30 included studies, 10 were prospective cohort studies and 20 were retrospective cohort studies. In addition, 13, 8, and 9 studies used the pRIFLE, AKIN, and KDIGO criteria for AKI diagnosis, respectively. Furthermore, Aydin et al. [11], Lee et al. [35], and Park et al. [41] not only reported the incidence rates in the general population but also stratified them by age.

**Table 1:** Characteristics of the studies included in the meta-analysis

Study/Year	Area/Design	Type of surgery	Time of recruitment	Criteria of AKI	n, M/F (%)	Age	AKI (%)
Amini, S 2017	Iran/PCS	CS for CHD	2013.03–2016.02	pRIFLE	519, 49.9/50.1	< 18 years	150 (28.9)
Aydin, SI 2012	USA/RCS	CS for CHD	2006.01–2009.11	pRIFLE	458, 52.4/47.6	< 18 years	234 (51.1)
					87, 52.9/47.1	< 1 month	53 (60.9)
					371, 52.3/47.7	1 month-18 years	181 (48.8)
Blinder, JJ 2017	USA/RCS	CS for CHD	2006.09–2012.05	AKIN	799, 53.8/46.2	0–36 ms	289 (36.2)
Fuhrman, DY 2019	USA/RCS	CS for CHD	2004.01–2015.12	KDIGO	699, 56.4/43.6	18–40 years	92 (13.2)
Garcia, RU 2020	USA/RCS	CS for CHD with CPB	2012.01–2017.12	pRIFLE	149, 65.1/34.9	14.9 (12.7–16.9) years	4 (2.7)
Gil-Ruiz Gil-Esparza 2014	Spain/RCS	CS for CHD with CPB	2008.01–2010.12	pRIFLE	409, 60.4/39.6	1 month-18 years	107 (26.2)
Hirano, D 2017	Japan/RCS	CS for CHD	2007.04–2013.08	pRIFLE	418, 62.0/38.0	5.0 (2.0–19.0) months	104 (24.9)
Huggins, N 2014	USA/RCS	CS for CHD	2007.01–2010.11	AKIN	113, NR/NR	< 18 years	46 (40.7)
Joffe, R 2018	Canada/PCS	CS for CHD with CPB	2013–2015	KDIGO	66, 51.5/48.5	5.9 (4.6–11.5) months	43 (65.2)
Kim, J 2020	USA/RCS	CS for CHD with CPB	2013.04–2018.05	AKIN	123, 49.6/50.4	≥18 years	52 (42.3)
Kimura, S 2018	Japan/RCS	CS for CHD with CPB	2013.12–2017.01	KDIGO	521, 53.4/46.6	< 72 months	205 (39.3)
Kumar, TK 2016	USA/RCS	CS for CHD with CPB	2010.01–2012.12	AKIN	102, 55.9/44.1	< 2 months	11 (10.8)
Kwiatkowski, DM 2017	USA/RCS	CS for CHD with CPB	2010.01–2013.12	KDIGO	118, 50.0/50.0	29 (21–39) years	42 (35.6)
Lee, SH 2017	Korea/RCS	CS for CHD with CPB	2011.04–2011.12	pRIFLE	135, 51.1/48.9	< 18 years	19 (14.1)
					13, NR/NR	< 1 month	4 (30.8)
					122, NR/NR	1 month-18 years	15 (12.3)
Li, S 2011	USA/PCS	CS for CHD	2007.07–2009.12	AKIN	311, 55.0/45.0	1 month-18 years	130 (41.8)
Madsen, NL 2017	Denmark/PCS	CS for CHD	1990.01–2010.12	KDIGO	382, 57.1/42.9	< 14 years	127 (33.2)
Mah, KE 2018	USA/RCS	CS for CHD	2010.10–2012.12	KDIGO	117, 59.8/40.2	< 30 days	66 (56.4)
Meersch, M 2014	Germany/PCS	CS for CHD with CPB	2013.07–2013.12	pRIFLE	51, 72.5/27.5	< 18 years	12 (23.5)
Miklaszewska, M 2013	Poland/PCS	CS for CHD with CPB	2006–2009	pRIFLE	47, 51.1/48.9	0.5–204 months	19 (40.4)
Park, SK 2016	Korea/RCS	CS for CHD	2012.01–2012.12	KDIGO	220, 55/45	10 days-18 years	92 (41.8)
					60, NR/NR	< 1 month	29 (48.3)
					160, NR/NR	1 month-18 years	63 (39.4)
Piggott, KD 2015	USA/RCS	CS for CHD	2010.05–2013.12	AKIN	95, 50.5/49.5	< 1 month	43 (45.3)
Ricci, Z 2012	Italy/PCS	CS for CHD with CPB	2010.06–2011.06	pRIFLE	160, 55/45	< 1 years	90 (56.3)
Ruf, B 2015	Germany/PCS	CS for CHD with CPB	2011.01–2011.08	pRIFLE	59, 59.3/40.7	< 1 years	28 (47.5)
Sugimoto, K 2016	Japan/PCS	CS for CHD with CPB	2010.07–2012.07	pRIFLE	376, 58/42	1 month-18 years	243 (64.6)
Tanyildiz, M 2017	Turkey/RCS	CS for CHD	2009.01–2011.10	pRIFLE	137, 53.3/46.7	1 month-18 years	84 (61.3)
Taylor, ML 2013	USA/RCS	CS for CHD	2009.01–2009.12	AKIN	693, 53/47	6 days-18 years	104 (15)
Toth, R 2012	Hungary/RCS	CS for CHD	2004.01–2008.12	pRIFLE	1510, NR/NR	< 18 years	481 (31.9)
Ueno, K 2020	Japan/RCS	CS for CHD	2010.05–2018.01	KDIGO	81, 60.5/39.5	< 1 month	57 (70.4)
Van Driest, SL2018	USA/RCS	CS for CHD	2008.07–2016.06	KDIGO	999, 50.0/50.0	1 month-18 years	503 (50.4)
Zheng, J 2013	China/PCS	CS for CHD with CPB	2010.11–2011.04	AKIN	693, 53/47	< 3 years	104 (15)

*n* number of participants; *M* male; *F* female; *AKI* Acute Kidney Injury; *PCS* prospective cohort study; *RCS* retrospective cohort study; *CS* cardiac surgery; *CHD* congenital heart disease; *CPB* Cardiopulmonary Bypass; *p-RIFLE* pediatric (Risk, Injury, Failure and Loss, and End-Stage) criteria; *AKIN* Acute Kidney Injury; *KDIGO* Kidney Disease Improving Global Outcomes criteria

### Assessment of methodological quality

The evaluation results of the methodological qualities of the included study are shown in Table 2. All the studies provided the source of information, inclusion and exclusion criteria, time period for recruiting patients, completeness of the data collection, and so on. However, information on confounding assessment, missing data handling, and clarifying follow-up was lacking in most of the included studies. Among the 30 included studies, 4 (13.3%) with scores of 8 were considered high-quality and the remaining 26, with scores between 5 and 7, were classified as moderate-quality. Overall, the methodologies of the included studies were of a moderate quality.

**Table 2:** The methodological quality evaluation of included literature based on AHRQ

Study	Year	A	B	C	D	E	F	G	H	I	J	K	Total Scores
Chen Ya-ke	2011	1	1	1	1	1	0	1	0	0	1	0	7
Dong Yu-fu	2005	1	1	1	1	0	1	1	0	0	1	0	7
Blinder, JJ	2017	1	1	1	1	0	0	1	0	1	1	1	8
Fuhrman, DY	2019	1	1	1	1	0	1	1	0	0	1	0	7
Garcia, RU	2020	1	1	1	1	0	0	1	0	0	1	1	7
Gil-Ruiz Gil-Esparza	2014	1	1	1	1	0	1	1	0	0	1	0	7
Hirano, D	2017	1	1	1	1	1	0	1	0	0	1	0	7
Huggins, N	2014	1	1	1	1	0	1	0	0	0	1	0	6
Joffe, R	2018	1	1	1	1	1	0	1	1	0	1	0	8
Kim, J	2020	1	1	1	1	0	1	0	0	0	1	0	6
Kimura, S	2018	1	1	1	1	1	0	1	0	0	1	0	7
Kumar, TK	2016	1	1	1	1	0	0	0	0	1	1	0	6
Kwiatkowski, DM	2017	1	1	1	1	0	1	1	0	0	1	1	8
Lee, SH	2017	1	1	1	1	1	0	1	0	0	1	0	7
Li, S	2011	1	1	1	1	0	0	1	0	0	1	0	6
Madsen, NL	2017	1	1	1	1	0	0	1	0	0	1	0	6
Mah, KE	2018	1	1	1	1	1	0	1	0	0	1	0	7
Meersch, M	2014	1	1	1	1	0	0	1	0	0	1	0	6
Miklaszewska, M	2013	1	1	1	1	0	1	1	0	0	1	0	7
Park, SK	2016	1	1	1	1	0	0	1	0	0	1	0	6
Piggott, KD	2015	1	1	1	1	1	0	1	0	0	1	0	7
Ricci, Z	2012	1	1	1	1	0	0	0	0	0	1	0	5
Ruf, B	2015	1	1	1	1	0	0	1	0	0	1	0	6
Sugimoto, K	2016	1	1	1	1	0	1	0	1	0	1	0	7
Tanyildiz, M	2017	1	1	1	1	0	0	1	0	0	1	0	6
Taylor, ML	2013	1	1	1	1	1	0	1	0	0	1	0	7
Toth, R	2012	1	1	1	1	0	0	1	0	0	1	0	6
Ueno, K	2020	1	1	1	1	0	0	1	0	0	1	0	6
Van Driest, SL	2018	1	1	1	1	0	1	1	0	0	1	1	8
Zheng, J	2013	1	1	1	1	0	0	1	0	0	1	0	6

A: Define the source of information (survey, record review); B: List inclusion and exclusion criteria for exposed and unexposed subjects (cases and controls) or refer to previous publications; C: Indicate time period used for identifying patients; D: Indicate whether or not subjects were consecutive if not population-based; E: Indicate if evaluators of subjective components of study were masked to other aspects of the status of the participants; F: Describe any assessments undertaken for quality assurance purposes (e.g., test/retest of primary outcome measurements); G: Explain any patient exclusions from analysis; H:Describe how confounding was assessed and/or controlled; I: If applicable, explain how missing data were handled in the analysis; J: Summarize patient response rates and completeness of data collection; K: Clarify what follow-up, if any, was expected and the percentage of patients for which incomplete data or follow-up was obtained

### Meta-analysis results of AKI in the patients with CHD

**Fig. 2:**
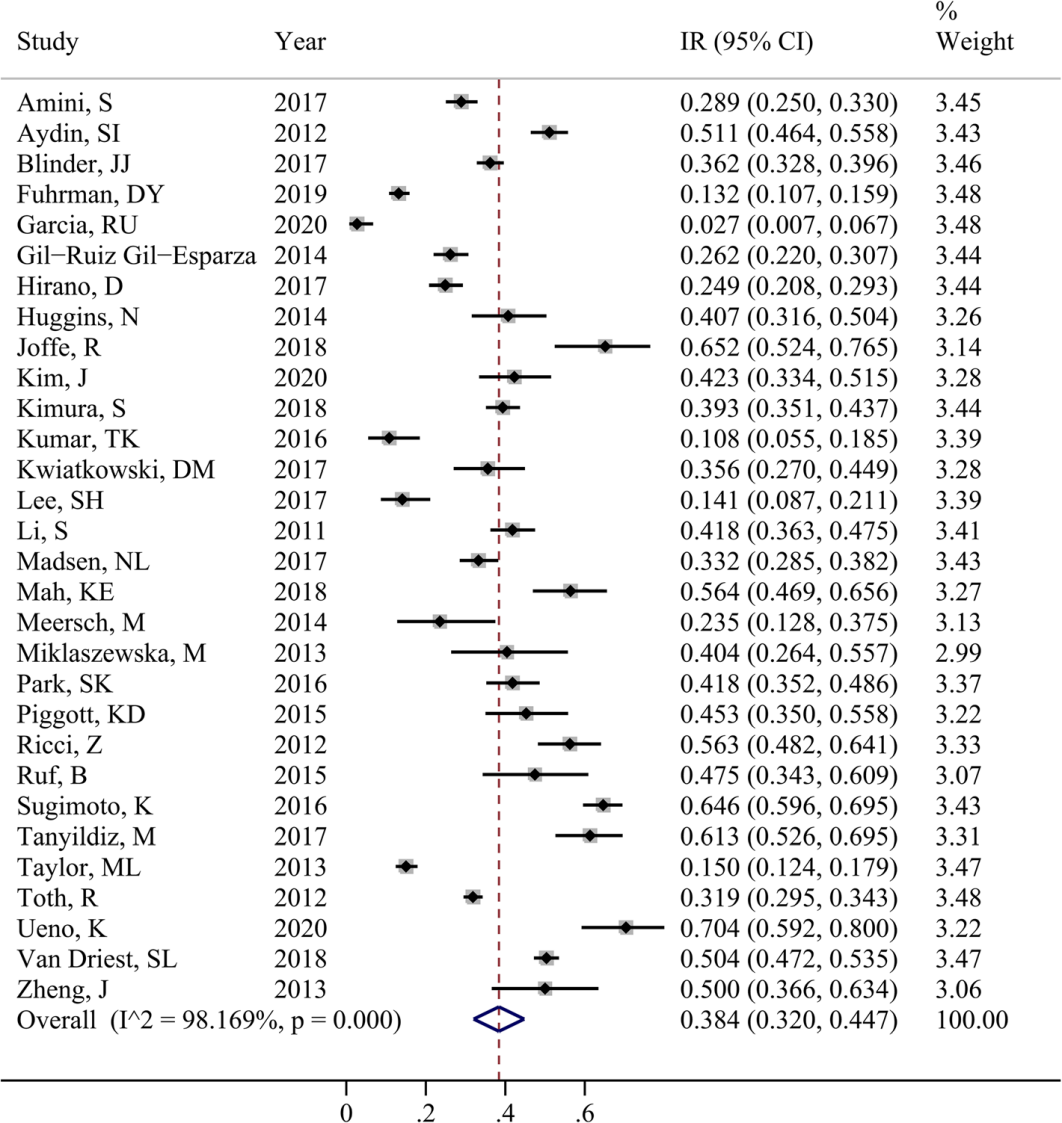
Forest plot of the included studies that assessed AKI incidence in the overall population. Each study is labeled with the name of the author and year of publication. The larger squares indicate a larger weight of the study in the calculation of the pooled estimate. The solid horizontal lines display the 95% CIs of the point estimates. The dashed vertical line represents the pooled estimate. **Note**: IR, incidence rate; CI, confidence interval. Abbreviations: AKI, acute kidney injury

Thirty studies [9–13, 26–50] reported the incidence rate of AKI. These studies were significantly heterogeneous (*I*^2^ = 98.169%, *P* < 0.001). The pooled estimated incidence rate was 38.4% (95% CI, 32.0–44.7%; Fig. 2) based on the random-effects model.

**Fig. 3:**
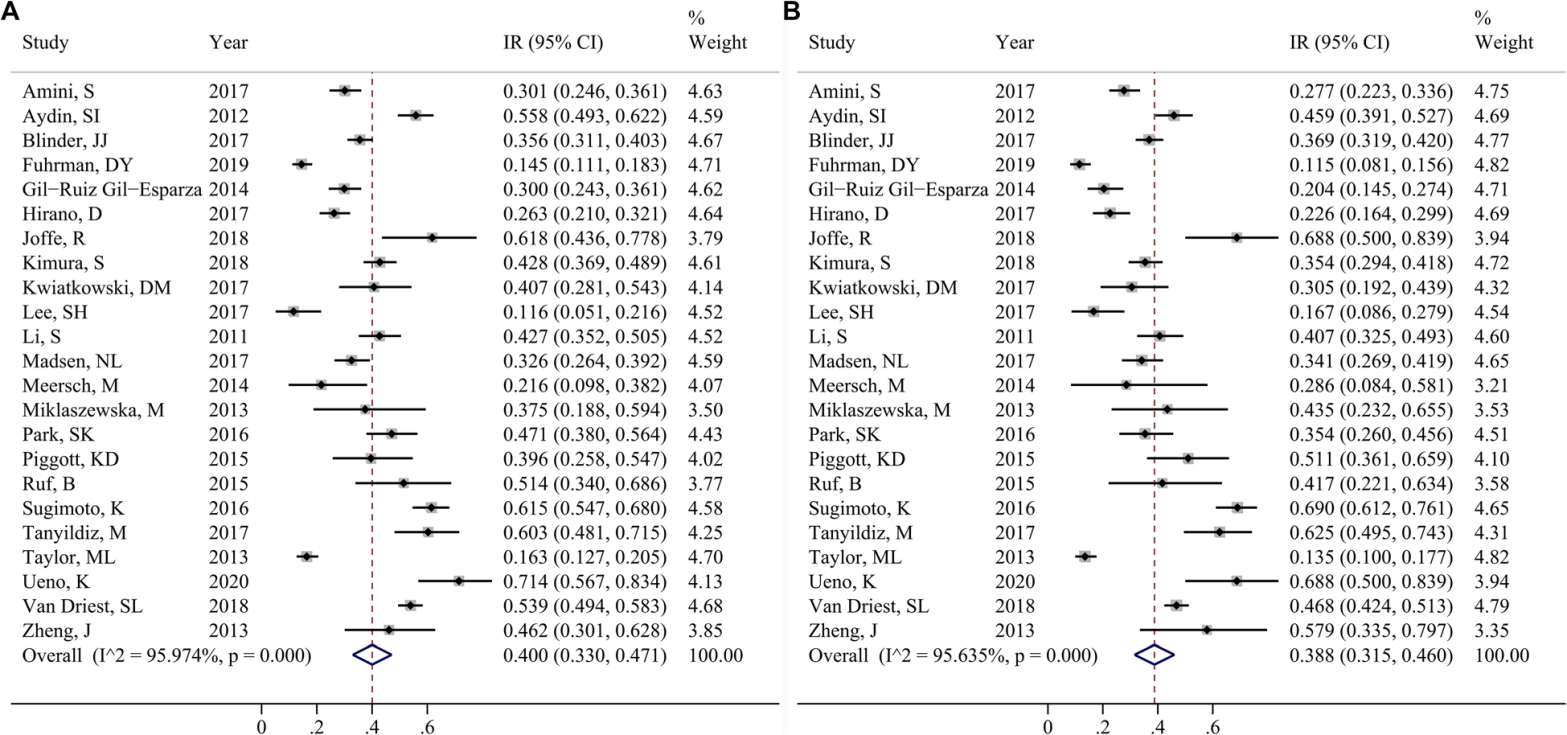
Forest plot of the included studies that assessed AKI incidence in male (**a**) and female (**b**) population. Each study is labeled with the name of the author and year of publication. The larger squares indicate a larger weight of the study in the calculation of the pooled estimate. The solid horizontal lines display the 95% CIs of the point estimates. The dashed vertical line represents the pooled estimate. **Note**: IR, incidence rate; CI, confidence interval. Abbreviations: AKI, acute kidney injury

Furthermore, the incidence rate of AKI among males was reported in 23 studies. The pooled incidence rate of AKI was 40.0% (95% CI, 33.0–47.1%; Fig. 3a). In addition, 23 studies reported the incidence rate of AKI in the female population. The pooled incidence rate was 38.8% (95% CI, 31.5–46.0%; Fig. 3b). However, no significant difference was observed between men and women (*P* < 0.05).

### Subgroup analysis

The subgroup analysis was conducted mainly using the following five aspects: study location, type of surgery, type of cohort, age, and AKI criteria (Table 3). The results of the subgroup analysis showed that the incidence rate of AKI was not significantly different between the subgroups stratified by study location, type of surgery, type of cohort, and criteria of AKI (*P* > 0.05). Notably, age was significantly associated with the incidence rate of AKI (*P* = 0.002). The pooled estimated incidence rates were 54.3% (95% CI, 45.2–63.3%) at age < 1 month, 41.6% (95% CI, 33.5–49.6%) at age 1 month to 18 years, 29.9% (95% CI, 20.9–38.9%) at age < 18 years, and 30.0% (95% CI, 9.60–50.5%) at age ≥ 18 years).

**Table 3:** Results of subgroup analysis

Outcomes	No. of studies	IR(95%CI)	Heterogeneity test		P-sub
			*P* I^2^ (%)		
Overall	30	0.384 (0.320, 0.447)	< 0.001	98.169	–
Location					0.291
Asia	9	0.436 (0.319, 0.554)	< 0.001	97.477	
Western	21	0.361 (0.287, 0.436)	< 0.001	98.268	
Type of surgery					0.694
CS for CHD	16	0.397 (0.324, 0.471)	< 0.001	98.024	
CS for CHD with CPB	14	0.368 (0.242, 0.494)	< 0.001	98.343	
Type of cohort					0.118
Prospective	10	0.451 (0.352, 0.550)	< 0.001	95.038	
Retrospective	20	0.352 (0.376, 0.427)	< 0.001	98.446	
Age					0.002
< 1 month	6	0.543 (0.452, 0.633)	0.002	73.164	
1 month-18 years	14	0.416 (0.335, 0.496)	< 0.001	96.927	
< 18 years	10	0.299 (0.209, 0.389)	< 0.001	97.854	
≥ 18 years	3	0.300 (0.096, 0.505)	–	–	
Criteria of AKI					0.471
pRIFLE	13	0.362 (0.255, 0.470)	< 0.001	98.584	
AKIN	8	0.348 (0.242, 0.453)	< 0.001	96.643	
KDIGO	9	0.447 (0.318, 0.576)	< 0.001	98.358	

*P-sub P* value of the difference between subgroups; −-, not available. *IR* incidence rate

### Publication bias

Publication bias was investigated using the Egger test, which revealed a significant publication bias between the studies (*P* = 0.007). Thus, the trim-and-fill method was applied to verify the stability of the pooled data. After correction by the trim-and-fill method, the incidence rate of AKI was 25.1% (95% CI, 18.5–31.7%). A significant difference was detected between the uncorrected and corrected pooled data (*P* = 0.004), which indicated that the effect of the small sample size may have excessively increased the risk of AKI after cardiac surgery in the patients with CHD.

## Discussion

In this meta-analysis, the pooled incidence rates of AKI in the patients with CHD were 38.4%,. However, the incidence rate of AKI showed no significant difference between men and women. In addition, the associations remained when the analyses were restricted to age.

Previous meta-analyses also indicated the incidence rates of AKI in other types of surgeries such as total hip arthroplasty (6.3%) [15], cardiac transplantation (47.1%) [16], and liver transplantation (40.8%) [51]. The pooled incidence rates of AKI in patients with CHD ranged from 30 to 45%, which was relatively higher than those in other types of surgery. The difference in surgery-related estimates could be attributed to the different clinical landscape and economic burdens. Typically, cardiopulmonary bypass (CPB) time, young age, preoperative AKI, preoperative mechanical ventilation, and perioperative peritoneal dialysis were known as risk factors for AKI development after a congenital heart surgery [26, 52, 53]. Furthermore, accumulating evidence suggests that AKI is a considerable risk factor of poor long-term outcomes, including chronic kidney disease and end-stage renal diseases [54]. Thus, these data indicate the need for better tools in predicting AKI and risk stratification of patients, as well as better preventive strategies.

Regarding the relationship between gender and the incidence rate of AKI, the incidence rate of AKI differs among patients with CHD. Li et al. demonstrated that the incidence of AKI was not associated with sex in 130 patients (57 women and 73 men) [36]. Among 82 patients with CHD, the difference between 57 men and 25 women was not significant in early AKI [29]. In accordance with our findings, sex exerts no influence on the incidence rate of AKI.

When stratified by age, our results indicated that the incidence rate of AKI significantly differed. Previous evidence showed that young children were more susceptible to AKI [55, 56]. A study concluded that the number of patients aged 1–12 months was significantly higher than that of patients aged > 12 months among 92 patients with AKI according to the KDIGO criteria [41]. Meanwhile, another study indicated that among 181 patients with AKI based on the AKIN staging system, 39% were aged 1 month to 2 years; 54%, 2–13 years; and 7%, 13–18 years, with significant differences in age [36]. This may be due to the fact that the intrinsically immature neonatal renal tubules may be more vulnerable to ischemic and inflammatory damage during heart surgery [36, 57]. Furthermore, complex cardiac anatomies and surgical repairs in children require longer CPB, which increases the risk of kidney injury [34, 36]. These results were consistent with those of the subgroup analysis in the present study. Consequently, the preliminary speculations that age was significantly associated with the incidence rate of AKI and that the incidence rate of AKI might be greater in patients aged < 1 month than in those aged 1 month to 18 years, < 18 years, and ≥ 18 years were confirmed.

In a subgroup analysis, the difference in location (Asian and Western countries) and type of surgery (coronary stenting [CS] and CS + CPB) showed no significant difference in the incidence rate of AKI. The estimates indicated that incidence rates in patients with AKI from Asian countries, except Japan, were much lower than those reported from Western countries (7–18%) [58]. Conversely, the results of this study showed that the research region had no influence on the incidence rate of AKI. These differences may be attributed to the study population, diagnostic testing capabilities, reliable epidemiological data, and available and accessible resources. Moreover, no strong association was observed between CPB duration and AKI development [26]. By contrast, a meta-analysis indicated that longer CPB duration was associated with higher risk of AKI development [59]. However, the addition of CPB could slightly reduce the incidence rate of AKI in the present study, but not statistically significantly. Inconclusive results were obtained because of the different study designs and limited sample size. Thus, further large-sample studies should be conducted to confirm these findings.

Delayed AKI diagnosis leads to increased morbidity and mortality rates. Therefore, patients at high risk of cardiac surgery-associated AKI need timely diagnosis using biomarkers and scoring systems [60]. Treatment is often limited to supportive care such as minimizing fluid overload and avoiding renal toxins, but may ultimately lead to the need for renal replacement therapy [52, 54]. Moreover, long-term care for AKI patients with CHD is recommended.

The several limitations in the present meta-analysis are as follows: First, the incidence rate of AKI was pooled using different criteria among studies. Owing to the differences in the included literature, the significance of the differences in the incidence of AKI cannot be scientifically compared. Second, because the results of the meta-analysis showed significant heterogeneity, its source could not be determined through a subgroup analysis. Third, the incidence rates of severe AKI requiring dialysis and the mortality of patients with AKI after cardiac surgery were not analyzed because of the limited data. Lastly, the small-study effects induced by the significant publication bias were assessed with the Egger test. The incidence of AKI in patients undergoing cardiac surgery should be verified in higher-quality studies.

## Conclusions

The incidence rate of AKI in patients with CHD was 38.4% and significantly associated with age. Moreover, well-designed studies with large sample sizes and higher quality are required to identify preventive strategies for AKI in patients with CHD.

## Supplementary information

**Additional file 1 Table S1** The detailed information relating to the retrieval steps and results of PubMed (The retrieval time: 20200424). **Table S2** The detailed information relating to the retrieval steps and results of Embase (The retrieval time: 20200424). **Table S3** The detailed information relating to the retrieval steps and results of The Cochrane library (The retrieval time: 20200424)

## Data Availability

The datasets used and analyzed in the present study are available from the corresponding author upon reasonable requests.

## Abbreviations

AKI: Acute kidney injury
CHD: Congenital heart disease
CI: Confidence interval
AKIN: AKI Network criteria
KDIGO: Kidney Disease Improving Global Outcomes criteria
