# Supplementary material for: A meta-analysis of the incidence rate of postoperative acute kidney injury in patients with congenital heart disease
Source: BMC Nephrol. 2020 Aug 17;21:350. doi: 10.1186/s12882-020-02005-2 (PMC7433101; doi:10.1186/s12882-020-02005-2)
Supplement: Supplementary file 1 — Additional file 1 Table S1 The detailed information relating to the retrieval steps and results of PubMed (The retrieval time: 20200424). Table S2 The detailed information relating to the retrieval steps and results of Embase (The retrieval time: 20200424). Table S3 The detailed information relating to the retrieval steps and results of The Cochrane library (The retrieval time: 20200424) [file 12882_2020_2005_MOESM1_ESM.docx]

**Sup Table 1** The detailed information relating to the retrieval steps and results of PubMed (The retrieval time: 20200424)

| Search | Query | Items found |
| --- | --- | --- |
| #1 | ("acute kidney injury"[MeSH Terms]) OR ("acute"[All Fields] AND "kidney"[All Fields] AND "injury"[All Fields]) OR ("acute kidney injury"[All Fields]) OR AKI[All Fields] OR ("acute"[All Fields] AND "renal"[All Fields] AND "failure"[All Fields]) OR ("acute renal failure"[All Fields]) OR ARF[All Fields] | 93334 |
| #2 | ("Congenit Heart Dis"[Journal] OR ("congenital"[All Fields] AND "heart"[All Fields] AND "disease"[All Fields]) OR "congenital heart disease"[All Fields]) OR ("chd"[All Fields]) OR ("heart defects, congenital"[MeSH Terms] OR ("heart"[All Fields] AND "defects"[All Fields] AND "congenital"[All Fields]) OR "congenital heart defects"[All Fields] OR ("congenital"[All Fields] AND "heart"[All Fields] AND "defects"[All Fields])) | 188507 |
| #3 | #1 AND #2 | 742 |

**Sup Table 2** The detailed information relating to the retrieval steps and results of Embase (The retrieval time: 20200424)

| Search | Query | Items found |
| --- | --- | --- |
| #1 | 'acute kidney injury'/exp OR 'acute kidney injury' OR AKI OR 'acute renal failure'/exp OR 'acute renal failure' OR ARF | 123177 |
| #2 | 'congenital heart disease'/exp OR 'congenital heart disease' OR CHD OR 'congenital heart defects' | 220580 |
| #3 | #1 AND #2 | 1418 |

**Sup Table 3** The detailed information relating to the retrieval steps and results of The Cochrane library (The retrieval time: 20200424)

| Search | Query | Items found |
| --- | --- | --- |
| #1 | MeSH descriptor: [Acute Kidney Injury] explode all trees OR (Acute Kidney Injury):ti,ab,kw OR (Acute Renal Failure):ti,ab,kw OR (AKI):ti,ab,kw OR (ARF):ti,ab,kw | 7421 |
| #2 | MeSH descriptor: [Heart Defects, Congenital] explode all trees OR (Congenital Heart Disease):ti,ab,kw OR (congenital heart defects):ti,ab,kw OR (CHD):ti,ab,kw | 6318 |
| #3 | #1 AND #2 | 81 |
